# The Toolbox for Uncovering the Functions of *Legionella* Dot/Icm Type IVb Secretion System Effectors: Current State and Future Directions

**DOI:** 10.3389/fcimb.2017.00528

**Published:** 2018-01-05

**Authors:** Gunnar N. Schroeder

**Affiliations:** Centre for Experimental Medicine, School of Medicine, Dentistry and Biomedical Sciences, Queen's University Belfast, Belfast, United Kingdom

**Keywords:** *Legionella*, Type IVb secretion system, Dot/Icm, effectors, toolbox, host targets, infection models, functional genomics

## Abstract

The defective in organelle trafficking/intracellular multiplication (Dot/Icm) Type IVb secretion system (T4SS) is the essential virulence factor for the intracellular life style and pathogenicity of *Legionella* species. Screens demonstrated that an individual *L. pneumophila* strain can use the Dot/Icm T4SS to translocate an unprecedented number of more than 300 proteins into host cells, where these, so called Icm/Dot-translocated substrates (IDTS) or effectors, manipulate host cell functions to the benefit of the bacteria. Bioinformatic analysis of the pan-genus genome predicts at least 608 orthologous groups of putative effectors. Deciphering the function of these effectors is key to understanding *Legionella* pathogenesis; however, the analysis is challenging. Substantial functional redundancy renders classical, phenotypic screening of single gene deletion mutants mostly ineffective. Here, I review experimental approaches that were successfully used to identify, validate and functionally characterize T4SS effectors and highlight new methods, which promise to facilitate unlocking the secrets of *Legionella*'s extraordinary weapons arsenal.

## Introduction

*Legionella pneumophila* was recognized as human pathogen in 1976 after a devastating outbreak of pneumonia, termed Legionnaires' disease, at an American Legion convention (Fraser et al., 1977; McDade et al., 1977). Investigations into the epidemiological and pathological mechanisms soon established that *L. pneumophila* is a ubiquitous, facultative intracellular pathogen of protozoa (Rowbotham, 1980), which, after inhalation, can also thrive in human alveolar macrophages. Key to exploiting phagocytic hosts is its ability to evade phago-lysosomal degradation (Horwitz, 1983a). Instead the bacteria create the *Legionella* containing vacuole (LCV) (Horwitz, 1983b), which shelters them from intracellular defenses and intercepts nutrients, supporting replication.

The defective in organelle trafficking/intracellular multiplication (Dot/Icm) Type IVb secretion system (T4SS) is critical for LCV biogenesis and intracellular replication (Berger and Isberg, 1993; Segal et al., 1998). It is located at the bacterial poles and, upon membrane contact, translocates proteins into host cells (Charpentier et al., 2009; Jeong et al., 2017), which manipulate cellular processes and are therefore called effectors. Although for many Icm/Dot-translocated substrates (IDTS) an actual effect on the host awaits demonstration, they will here collectively be referred to as effectors.

Facilitated by the genome sequences of prototype *L. pneumophila* strains (Cazalet et al., 2004; Chien et al., 2004) screens for T4SS substrates established that each strain translocates more than 300 proteins (Nagai et al., 2002; Luo and Isberg, 2004; De Felipe et al., 2005, 2008; Kubori et al., 2008; Burstein et al., 2009; Huang et al., 2011; Zhu et al., 2011; Lifshitz et al., 2013). Comparative genomics of an increasing number of *L. pneumophila* isolates and more than 38 *Legionella* spp. showed that, while sharing the Dot/Icm T4SS, extensive diversity in the effector arsenals exists (Schroeder et al., 2010; Gomez-Valero et al., 2014; Burstein et al., 2016). Only 7 proteins of an estimated 608 orthologous groups of effectors across the genus are conserved in all species (Burstein et al., 2016).

Despite advances in our understanding about some effectors (Finsel and Hilbi, 2015; So et al., 2015; Qiu and Luo, 2017), we still lack knowledge about the functions of the majority. Deciphering their functions is challenging, as effectors are a heterogeneous group with limited homology to characterized proteins. This mini-review summarizes methods that were employed to characterize Dot/Icm T4SS effectors and highlights additional methods that could help uncovering the weapons which *Legionella* spp. hold in their arsenals.

## Characteristics of Dot/Icm T4SS effectors

**Figure 1:**
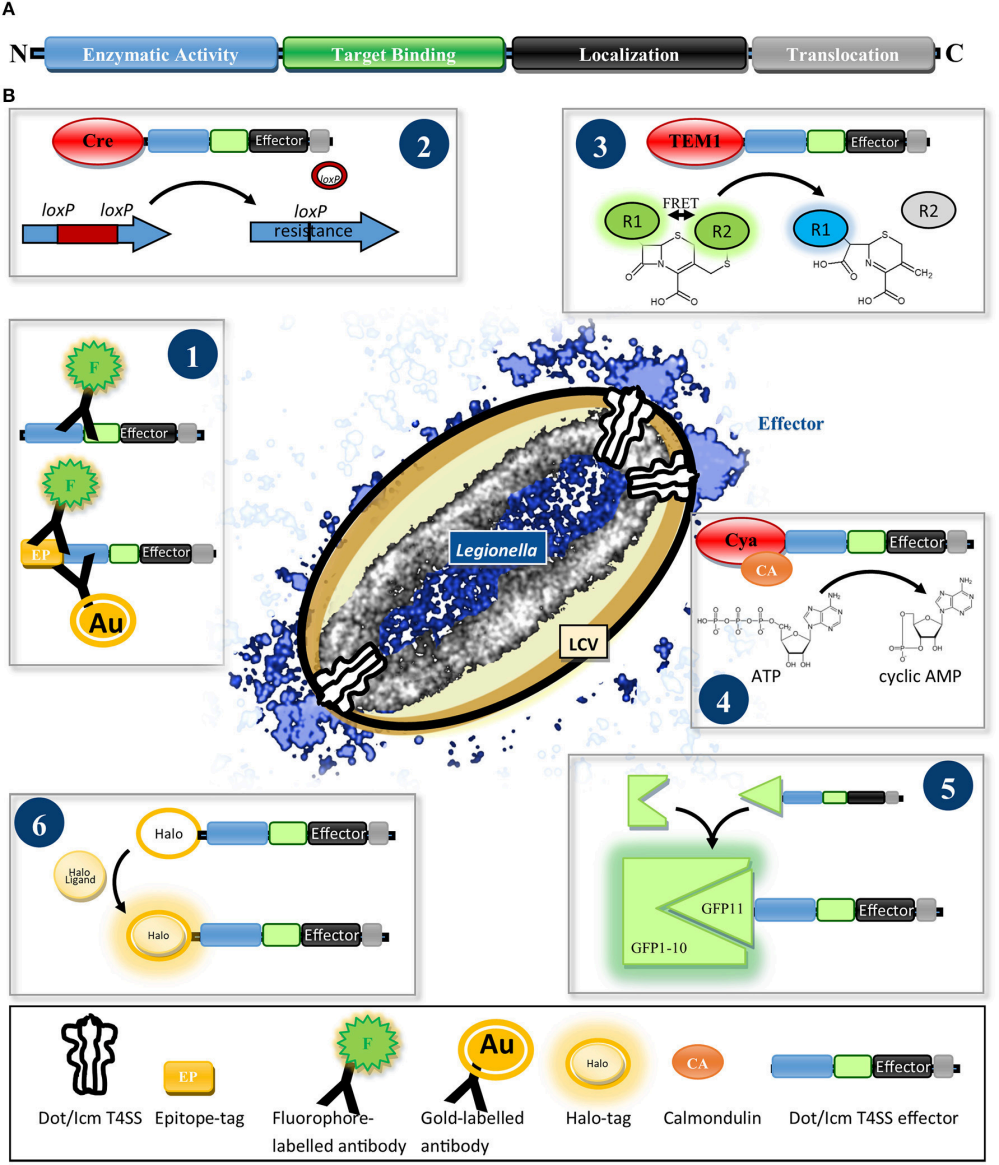
**(A)** Scheme of the typical architecture of Dot/Icm T4SS effectors. Effectors often show a modular structure consisting of a translocation signal and localization, target binding and enzymatic activity domains. **(B)** Reporter systems for determining the translocation and localization of Dot/Icm T4SS effectors. (1) Fluorophore- or gold particle-conjugated antibodies specific for an effector or an epitope tag are used to detect the effector in host cell lysates by immunoblot or visualize it by immunofluorescence- or electron microscopy. (2) Cre/*loxP* recombinase system: After delivery of Cre-effector fusions into recipient cells the recombinase removes a loxP-flanked disruptor cassette from a gene reporter conferring antibiotic resistance. (3) β-lactamase (TEM-1) assay: Translocation of TEM-1-effector fusions results in hydrolysis of a green fluorescent β-lactam FRET substrate, separating FRET donor and acceptor, generating a blue fluorescent product. (4) Calmodulin-dependent adenylate-cyclase (Cya) assay: Upon arrival of a Cya-effector fusion in the host, Cya gets activated by binding calmodulin and turns over ATP to cyclic AMP, which can be quantified by ELISA. (5) Split-GFP reporter system: Effectors are fused to the small (GFP11) fragment of a split GFP. Upon delivery into cells expressing the non-fluorescent large (GFP1-10) GFP fragment, spontaneous reassembly of effector-fused GFP11 and GFP1-10 occurs, restoring fluorescence emission which can be visualized by fixed or live imaging fluorescence microscopy. (6) Halo-tag reporter: After translocation into the host the Halo-tagged effector can be ligated with versatile fluorophores for detection by conventional, super-resolution- or electron microscopy.

Work over the past 15 years revealed several characteristics of effectors. A translocation signal, which directs them to the T4SS, is commonly found in the C-terminus (Nagai et al., 2005). It consists of a pattern of 20–35 amino acids with specific biophysical properties, e.g., small polar and/or charged residues, and can include a so called E-Block motif encompassing several glutamic acid residues (Nagai et al., 2005; Kubori et al., 2008; Burstein et al., 2009; Huang et al., 2011; Lifshitz et al., 2013). Some effectors comprise an additional internal export signal (Cambronne and Roy, 2007; Jeong et al., 2015). Many effectors are large (>100 kDa), with modular architecture (Figure 1A), consisting of different functional domains, e.g., localization, target binding, and enzymatic activity domains. Most prominent feature, which facilitated the discovery of the first effector RalF, is the occurrence of domains with striking homology to eukaryotic proteins (Nagai et al., 2002; Cazalet et al., 2004; De Felipe et al., 2005; Gomez-Valero et al., 2011).

Integration of these characteristics and parameters, such as regulatory motifs, in machine-learning approaches enabled prediction algorithms. Several programs are available (Meyer et al., 2013; Zou et al., 2013; An et al., 2016). Dot/Icm effector-focused algorithms were applied to 38 *Legionella* spp., revealing not only 608 orthologous groups of effectors, but also 99 frequently-occurring domains, which facilitate the identification of new effectors (Burstein et al., 2009, 2016; Lifshitz et al., 2013).

## Probing translocation and localization

Several assays for the validation of T4SS-mediated transport exist (Figure 1B). The visualization of endogenous effectors in host cells using antibodies and immunofluorescence (IF) or electron microscopy (EM) is the gold standard to infer physiologically accurate information (Figure 1B.1) and was achieved for a few effectors, e.g., SdeA, LidA, RidL, SidC, SidM, RalF, (Nagai et al., 2002; Luo and Isberg, 2004; Bardill et al., 2005; Machner and Isberg, 2006; Finsel et al., 2013). An antibody against SidC was used to visualize the reconstitution of translocation of a SidC variant lacking its translocation signal by fusion to putative effectors (Vanrheenen et al., 2006; Huang et al., 2011).

Alternatively, effectors were detected in host cell extracts by immunoblot (Vanrheenen et al., 2006; Lin et al., 2015), which in combination with fractionation steps to isolate organelles or LCVs, also informed about their subcellular localization (Ivanov et al., 2010; Hoffmann et al., 2014a; Lin et al., 2015).

As many effectors seem to be of low abundancy, several assays employ overexpression and exploit that the T4SS tolerates reporter domains fused to the N-terminus of effectors, if these do not fold rapidly into rigid structures (Amyot et al., 2013). One or multiple epitope tags [e.g., M45 (Weber et al., 2006), 4xHemagglutinin (HA) (Dolezal et al., 2012), 13xMyc (Viner et al., 2012) or 3xFlag (Isaac et al., 2015)] were employed to detect translocated effectors.

An early screen using an enzymatic reporter domain measured restoration of an antibiotic resistance gene by the Cre/*loxP* recombinase after T4SS-mediated translocation of Cre-effector fusions from a *Legionella* donor into bacterial recipients (Figure 1B.2) (Luo and Isberg, 2004). However, the β-lactamase TEM-1 and the calmodulin-dependent adenylate-cyclase domain of *Bordetella pertussis* toxin Cya are the most frequently used enzymatic reporters (Figures 1B.3, 4), providing high sensitivity by enzymatic signal amplification (TEM1: cleavage of a β-lactam fluorescence resonance energy transfer (FRET) sensor; Cya; generation of cyclic AMP) (Chen, 2004; Nagai et al., 2005; De Felipe et al., 2008).

Despite localization of several effectors by IF, SidC is the only imaged by super-resolution microscopy (Naujoks et al., 2016) and no live imaging data tracking *Legionella* effectors during infection exists. Lack of fluorescent protein tags compatible with T4SS-mediated translocation might account for this. Split fluorescent proteins, used for *Salmonella* T3SS effectors (Figure 1B.5) (Van Engelenburg and Palmer, 2010) and new enzyme-tags (Figure 1B.6) (Halo-, Snap and Clip-tags), which self-label with fluorophores that are suitable for live-, super-resolution- and electron-microscopy (Bottanelli et al., 2016; Liss et al., 2016), are promising tools to reveal the dynamics and distribution of effectors on a nanoscale.

## Infection models

*Legionella* effectors target fundamental processes, conserved between protozoa and mammals, resulting in a range of infection models, which, despite similarities, have each strengths, and weaknesses. *Hartmannella vermiformis, Naegleria* spp. and in particular *Acanthamoeba castellanii* and *Dictyostelium discoideum* are frequently used environmental hosts (Rowbotham, 1980; Newsome et al., 1985; Fields et al., 1990; Moffat and Tompkins, 1992; Solomon and Isberg, 2000; Hoffmann et al., 2014b). Requirements for specific effector subsets in different protozoa vary and are more stringent than in macrophages (O'Connor et al., 2011), making protozoa indispensable to study the evolutionary pressures behind the acquisition of effectors. To analyze the interaction of *Legionella* with macrophages, various mammalian [e.g., U937 (Pearlman et al., 1988), HL-60 (Marra et al., 1990), THP-1, Raw264.7 (Cirillo et al., 1994), J774 (Husmann and Johnson, 1992), M-HS (Matsunaga et al., 2001)] and insect [S2 and Kc167 (Dorer et al., 2006; Sun et al., 2013)] cell lines served as models. Moreover, non-phagocytic cells [e.g., HEp-2 (Cirillo et al., 1994), A549 (Mousnier et al., 2014), HeLa (Finsel et al., 2013), HEK293 (Losick et al., 2010), CHO (McCusker et al., 1991; Kagan and Roy, 2002)], with optional ectopic-expression of Fcγ-receptor to boost the invasion efficiency of *Legionella*, were employed. To evaluate the relevance of findings for human disease, differences in patterns of protein family expansion, e.g., Rab GTPases (Klöpper et al., 2012), and innate immune signaling, e.g., inflammasome activation (Krause and Amer, 2016), between cell lines, mice and humans need to be considered. Ultimately, results need validation in primary macrophages and *in vivo* models that approximate the complexity of the human immune system.

Insects such as *Drosophila melanogaster* (Kubori et al., 2010) and *Galleria mellonella* (Harding et al., 2012, 2013b; Aurass et al., 2013) mount innate immune responses and represent straight-forward infection models. Tests in mammals, e.g., guinea pigs, rats, rhesus monkeys, and marmosets, showed that guinea pigs develop disease similar to humans (Baskerville et al., 1983; Davis et al., 1983). Mice, with exception of A/J mice, which are defective in an NAIP5-dependent inflammasome response to flagellin, are resistant to *Legionella* (Brieland et al., 1994). Nevertheless, because of the wealth of engineered mouse strains, infections of A/J mice with wild-type or non-permissive mice with flagellin-deficient *Legionella* have become the predominant *in vivo* models and gave important insight into effector and immune biology (Brown et al., 2016). In the future, humanized mice (Walsh et al., 2017) and *ex vivo* human lung tissue models (Jäger et al., 2014) will improve our capabilities to define roles of effectors in human infection.

## Genetics approaches to determine effector functions

*Legionella* is amenable for gene deletion by homologous recombination (Merriam et al., 1997; Bryan et al., 2011; O'Connor et al., 2011) and mutagenesis with transposons (Ott, 1994; Pope et al., 1994; Edelstein et al., 1999; O'Connor et al., 2011). Assays recording intracellular growth by colony counting or continuously, using fluorescent or bioluminescent strains, are established (Coers et al., 2007; Tiaden et al., 2013; Schroeder et al., 2015). Mixed infection competition experiments measuring performance of a wild type vs. a mutant strain achieved better resolution of differences in virulence in some cases (Ensminger et al., 2012; Finsel et al., 2013; Harding et al., 2013b). However, attenuation of strains lacking single effectors was rarely observed. Some effectors might be dispensable in a specific host; but *Legionella* also achieves resilience by deploying families of paralogue effectors, which seem functionally redundant (Cazalet et al., 2004; Chien et al., 2004).

**Figure 2:**
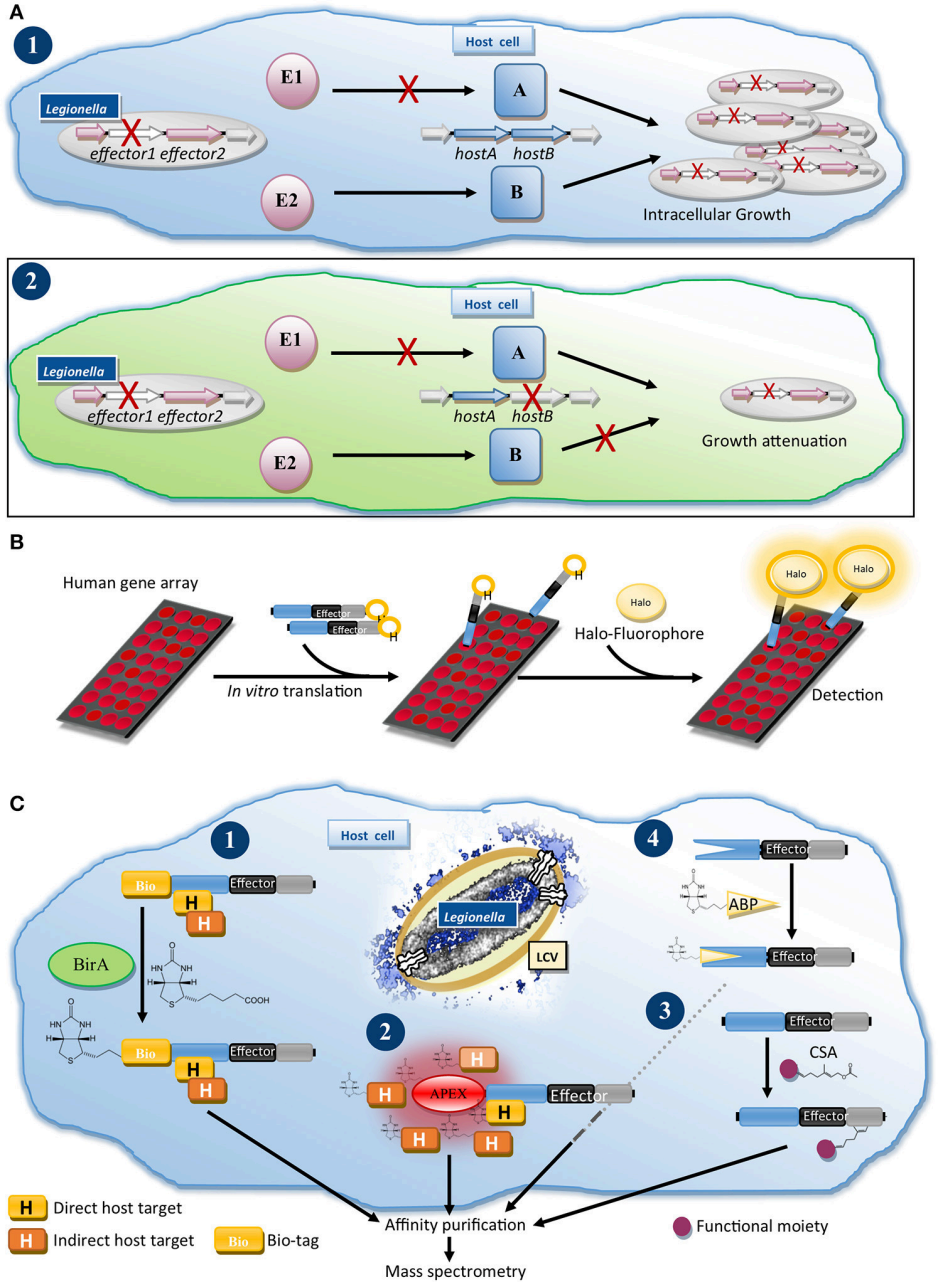
Genetics and proteomics methods for the functional characterization of Dot/Icm T4SS effectors. **(A)** Insertional mutagenesis and depletion (iMAD) disentangles the complex network of effector-host manipulations: (1) Characterization of a *Legionella* mutant lacking a single effector (E1), which acts through host protein A, does not result in reduced intracellular growth, because a second effector (E2) induces a redundant process through host protein B. (2) Following the iMAD strategy, screening of single effector mutants in host cells, which are also depleted for host factors, eliminates redundant pathways, resulting in attenuation of the strain. **(B)** Nucleic Acid-Programmable Protein Array (NAPPA) for profiling of host cell targets of effectors: Human genes are printed as array on slides and translated *in vitro*. Recombinant Halo-tagged effector is added and after washes reacted with a fluorophore-ligand for the Halo-tag, allowing detection of the human proteins, which bound and retained the effector on the array. **(C)** Proteomics approaches for the characterization of effectors: (1) BirA/Bio-tag system: Bio-tagged effector is translocated into biotin ligase BirA expressing cells leading to biotinlylation of the Bio-tag. After optional cross-linking the bioinylated effector and bound host proteins are isolated by tandem-affinity purification for interactome analysis by MS. (2) Proximity biotinylation: Translocation of an effector fused to e.g., the peroxidase APEX or a promiscuous biotin ligase (BioID) results in biotinylation of host proteins in the proximity of the effector, which can be processed as for (1) to identify potential interactors. (3) Identifying post-translational modifications (PTMs): Infected cells are infused or metabolically-labeled with a chemical substrate analog (CSA) for a PTM-catalyzing enzyme. Host proteins and effectors, which are modified with the CSA, can be isolated after ligation of an affinity handle such as biotin to the CSA and characterized by MS. (4) Profiling the enzymatic activities of effectors with activity-based probes (ABPs): Infected cells or lysates are treated with a chemical ABP, which irreversibly binds to a specific enzyme class and contains or allows addition of an affinity handle. Effectors, which possess such enzymatic activity, react with the probe, are isolated and identified by MS.

To reduce the complexity of the effector network, O'Connor and Isberg developed two genetic approaches. Insertional mutagenesis and depletion (iMAD, Figure 2A) is based on the combinatorial screening of effector deletion mutants for intracellular growth in hosts, which are also host factor depleted (O'Connor et al., 2012; O'Connor and Isberg, 2014). Additive or compensatory effects of the lack of an effector and a host factor are monitored and interrogated using computational clustering and network analysis, grouping effectors with similar profiles and predicting functional redundancy.

In a second approach five genomic regions were deleted to create a minimized genome strain lacking 31% of effectors (O'Connor et al., 2011). This strain grows normally in macrophages; but is attenuated in protozoa, underlining the importance of examining several infection models. Subsequently, the minimized genome strain and intermediates lacking subsets of the genomic regions proved to be valuable tools to link Dot/Icm T4SS dependent phenotypes to a chromosomal region and, through gene-by-gene screening, to individual effectors (Choy et al., 2012; Arasaki et al., 2017; Kotewicz et al., 2017). Deletion of additional effectors could generate even more powerful strains for loss-of-function or gain-of-function experiments, in which the perturbation of host processes by individual effectors can be dissected.

## Heterologous expression systems for phenotypic analysis

Alternatives to investigating effectors during infection rely on heterologous expression and delivery. These are often technically and analytically less complex, but do not reflect physiological concentrations, microenvironment of delivery and the effects of other effectors. Microinjection of recombinant effectors, e.g., SetA (Jank et al., 2012), offers excellent control of concentration and timing of injection, enabling the characterization of toxic effectors; however requires protein purification and a microinjector. Relinquishing the tight control over the delivery, but reducing technical requirements, microbial microinjection exploits a *Yersinia enterocolitica* strain with functional type III secretion system (T3SS), but lacking effectors (Wölke et al., 2011), to deliver individual Dot/Icm effectors (Rothmeier et al., 2013). The suitability of this approach for a wide range of T4SS effectors still needs confirmation.

Ectopic-expression in mammalian cells remains the workhorse to assess effector-induced modulation of host processes and subcellular targeting by co-localization with organelle markers. Numerous studies exist. Libraries containing up to 275 effectors for viral transduction or transfection were used to screen for effectors, which modulate, e.g., caspase activation (Zhu et al., 2013), the cytoskeleton (Liu et al., 2017), translation (Barry et al., 2013), or NF-kB activation (Ge et al., 2009; Losick et al., 2010).

*Saccharomyces cerevisiae* is an important tool to study effectors (Popa et al., 2016). Phenotypic screens identified *Legionella* effectors that subvert endosomal trafficking or are cytotoxic for yeast (Campodonico et al., 2005; Shohdy et al., 2005). The availability of yeast gene deletion and overexpression strain collections (Gelperin et al., 2005; Sopko et al., 2006; Giaever et al., 2014) bears particular potential. These have proven useful to test enzymatic activities of effectors, e.g., LegS2 or SidP, in functional complementation assays (Degtyar et al., 2009; Toulabi et al., 2013) and to profile synergistic and antagonistic genetic interactions between yeast and effector genes, allowing to infer affected pathways (Viner et al., 2012). Moreover, screening of overexpressed host proteins or effectors for suppression of effector-induced toxicity toward yeast identified host targets, effectors pairs with antagonistic activities and, so called meta-effectors, which regulate other effectors (Tan and Luo, 2011; Tan et al., 2011; Guo et al., 2014; Urbanus et al., 2016).

## Identification of protein targets

Dissecting the molecular mechanisms underlying effector-induced phenotypes often requires the identification of host targets. Yeast two-hybrid screening is a powerful method to identify protein-protein interactions and was used for several effectors (Banga et al., 2007; Lomma et al., 2010; Harding et al., 2013a; Michard et al., 2015). Similarly, pull-down of interactors from host cell lysates using purified effector or co-immunoprecipitation (Co-IP) from cells ectopically expressing an effector bait were frequently used (Machner and Isberg, 2006; Price et al., 2009; Finsel et al., 2013; Urbanus et al., 2016). In a cell-free assay system, the Nucleic Acid-Programmable Protein Array (NAPPA, Figure 2B) (Yu et al., 2015), human bait gene arrays are translated *in vitro*, exposed to Halo-tagged effector and bound effector detected by ligation of a fluorophore to the Halo-tag. This system, circumventing protein isolation, promises to reveal a global view of interactors.

Despite their proven value, all above-mentioned *in vitro* and heterologous expression methods struggle with the identification of false-positive and -negative targets, because they do not reflect the unique proteomic landscape which an effector experiences when injected at the LCV membrane into a cell that responds to the infection and is manipulated by hundreds of effectors.

We established a method to determine the interactomes of effectors during infection (Figure 2C.1) (Mousnier et al., 2014). *Legionella* expressing an effector fused to a tandem-affinity tag including a biotinylation site (Bio-tag), are used to infect cells expressing *Escherichia coli* biotin ligase BirA. The translocated effector is biotinylated, allowing isolation of effector-host target complexes for analysis by mass spectrometry (MS). Using this approach, we identified new interactors of PieE and profiled the infection-relevant interactions of the promiscuous Rab GTPase-binding effectors SidM and LidA (Mousnier et al., 2014; So et al., 2016).

Exciting prospects for effector target discovery arise from the development of proximity-biotinylation systems (Figure 2C.2). These rely on promiscuous biotinylation of proteins in proximity of engineered BirA (Roux et al., 2012) or the peroxidase APEX2 (Hung et al., 2016) followed by characterization of biotinylated targets by MS. Translocation of T3SS effector-APEX2 fusions by *Chlamydia* was recently described (Rucks et al., 2017) suggesting that this could be adopted for T4SS effectors.

## Profiling post-translational modifications (PTMs) and enzymatic activities

Effectors exploit host proteins as receptors (Gaspar and Machner, 2014) and subvert their functions, which is often achieved by post-translational modification (PTM) (Michard and Doublet, 2015). The discovery of the phosphocholination activity of AnkX and phosphoribosyl-ubiquitin ligase activity in SdeA illustrated that careful analysis of protein targets by MS is key to identify new PTMs (Mukherjee et al., 2011; Bhogaraju et al., 2016; Qiu et al., 2016). PTM specific antibodies were used e.g., to study effector-mediated phosphorylation or histone modifications (Ge et al., 2009; Rolando et al., 2013). This can be complemented by autoradiography assays using radioactive substrates, which excel in sensitivity, and are employed to study, e.g., AMPylation (Neunuebel et al., 2011; Tan and Luo, 2011) or glycosyltransferase effectors (Jank et al., 2012). Non-radioactive chemical substrate analogs (CSAs), which can be functionalized to visualize and isolate modified proteins, were developed for several PTMs (Grammel et al., 2011; Lu et al., 2012; Fischle and Schwarzer, 2016). CSAs can also reveal PTMs on effectors, as demonstrated for the post-translational lipidation of effectors (Figure 2C.3) (Ivanov et al., 2010; Lin et al., 2015; Schroeder et al., 2015). CSAs enable profiling of PTMs on proteome level from cell extracts, living cells or, as shown for SidM-mediated AMPylation, on NAPPA arrays (Yu et al., 2015), promising global overviews of PTMs at a coverage similar to the *Legionella*-shaped ubiquitinome (Ivanov and Roy, 2013; Bruckert and Abu Kwaik, 2015).

The discovery of new enzymatic activities is challenging as small molecules, e.g., ATP or lipids, can be substrates and/or effectors not necessarily target host proteins. Bioinformatic analysis to identify homologous enzymes and catalytic motifs is critical to find leads (Watson et al., 2005) for focused enzymatic assays, as exemplified by LpdA (lipolysis, Schroeder et al., 2015), SidF (phosphate release, Hsu et al., 2012) or LncP (nucleotide transport, Dolezal et al., 2012). Biophysical methods, e.g., differential scanning fluorimetry, allow screening of ligands (Ciulli, 2013).

For the identification of enzymes-of-interest, e.g., redundant effectors, in the *Legionella* proteome activity-based probes (ABPs) offer a solution (Figure 2C.4). ABPs are typically small molecules that irreversibly react with a specific enzyme class and functionalized to allow purification of modified enzymes for MS analysis. ABPs are available for many enzymes including the ubiquitin-conjugation and chromatin-modifying machineries (Willems et al., 2014; Fischle and Schwarzer, 2016; Hewings et al., 2017). ABPs will help to disentangle the redundancy problem, probe for eukaryotic-like enzymes and assign functions in new *Legionella* isolates.

## Synthesis

Deciphering the functions of thousands of effectors is a formidable challenge; however new genetics tools and a rapidly growing number of chemical biology and proteomics methods provide a well-suited toolbox to reveal fascinating new mechanisms of host manipulation by *Legionella*.

## Author contributions

The author confirms being the sole contributor of this work and approved it for publication.

### Conflict of interest statement

The author declares that the research was conducted in the absence of any commercial or financial relationships that could be construed as a potential conflict of interest.
